# Nationwide Etoricoxib Injection Clinical Experience (NICE): Real-World Evidence in Indian Patients

**DOI:** 10.7759/cureus.54020

**Published:** 2024-02-11

**Authors:** Umesh Shetty, Pramod Neema, S. Muthu, Divya Bhojwani, Sameer Muchhala

**Affiliations:** 1 Orthopaedics, Axis Multispecialty Hospital, Mumbai, IND; 2 Orthopaedics, Unique Super Speciality Hospital (Neema Hospitals Pvt. Ltd), Indore, IND; 3 Orthopaedics, Mallige Hospital, Bengaluru, IND; 4 Medical Affairs, Zydus Healthcare Ltd, Mumbai, IND; 5 Medical Affairs, Zydus Lifesciences Ltd, Mumbai, IND

**Keywords:** analgesic, emergency, trauma, ra, oa, etoricoxib, nsaid, acute pain, indian population, real-world study

## Abstract

Introduction

Pain is a major health issue globally. Etoricoxib, a highly selective COX-2 inhibitor, given orally, has been found to be efficacious and safe in the management of acute and chronic pain. Oral etoricoxib has been extensively studied; however, there is a lack of research exploring the use of etoricoxib via alternative routes, specifically intramuscular (IM) injection. This study aimed to evaluate the effectiveness and safety of an innovative and novel formulation of IM etoricoxib injection 90 mg/mL in the management of patients with acute pain in India.

Method

This was a real-world, multicenter, retrospective, observational study to investigate the effectiveness and safety of IM etoricoxib injection in the management of patients with acute pain in India (outpatient setting). The clinical data of 383 patients from 42 centers across India were collected from November 2022 to April 2023. Following approval from the site investigator, comprehensive patient-level information encompassing demographic and clinical variables as well as comorbidities was collected and entered into a case report form. Approval from the Independent Ethics Committee (IEC) and Institutional Review Board (IRB) was sought. The safety and effectiveness at 30 minutes and 60 minutes of IM etoricoxib injection were then analyzed.

Results

Among the studied patients, etoricoxib was used for the management of knee arthritis, fracture, post-traumatic pain, postoperative cases, acute back pain, back injury, torn ligament, and muscle strain and sprain. Of 383 patients, 98.17% had moderate-to-severe pain at baseline on the visual analogue scale (VAS) (0 being no pain and 10 being severe unbearable pain). The percentage of patients with severe pain reduced to only 6.78% and 4.17% at 30 minutes and 60 minutes, respectively. The improvement in the VAS score was statistically significant from baseline to 30 minutes and 60 minutes, and at 60 minutes compared to 30 minutes (p < 0.0001). More than half the patients (56.91%) had no pain at the site of the injection. Most of the doctors (70.23%) opined that the IM etoricoxib injection was better than their currently used NSAID injections for pain relief. Only 12.79% of patients required rescue analgesia post-IM etoricoxib injection. IM etoricoxib injection was well tolerated as 98.69% of the patients did not experience or report any adverse events post-IM injection.

Conclusion

This real-world, multicenter, retrospective, observational study across India demonstrated that the innovative and novel formulation of etoricoxib (90 mg/mL IM injection) was effective and well-tolerated in the management of acute pain. Overall, this study provides valuable insights into the real-world effectiveness and safety of IM etoricoxib injections, suggesting it could be a promising therapy for the management of acute pain for optimal patient benefit.

## Introduction

Pain is a major public health issue worldwide [1]. It is a very unpleasant sensation that makes communication with caregivers difficult for the patient. Pain is reported to be the most common cause for consultation in the emergency department [2]. Globally, in adults aged over 25 years, between 9.9% and 50.3% of the population experience pain [3]. Pain negatively impacts the quality of life. In a recent study, Goyal et al. observed that among Indian patients with pain, the average quality of life score was 81.6 versus 85.2 among those without pain. Evidence suggests that pain has a severe impact on most segments of life, including sleep, the ability to perform household activities, exercise, and walking [1].

Depending on the duration, pain is classified into two types: acute and chronic. Acute pain lasts for hours, days, or weeks, whereas chronic pain persists for months, years, or a lifetime [1]. Acute pain can be the result of tissue damage, surgical procedures, or a short disease process [4]. In hospital and ambulatory settings, acute pain is prevalent in 30% to 80% of the patients. Moreover, untreated pain can lead to chronic pain, which leads to a longer hospital stay and higher morbidity [5]. Unlike acute pain, chronic pain worsens over time and increases in severity. Chronic pain can be the result of disease processes (cancer, arthritis, etc.), unresolved injury over a long time, or surgeries like thoracotomy and mastectomy [4]. Despite its high prevalence, pain is often managed inadequately because the prescription of analgesic treatment is suboptimal, the analgesic is inappropriate, or its dose [2]. Additionally, in India, frequent use of polypharmacy along with inadequate knowledge significantly impacts analgesic efficacy and leads to concerns regarding tolerability [5].

Nonsteroidal anti-inflammatory drugs in the management of pain

Paracetamol or nonsteroidal anti-inflammatory drugs (NSAIDs) are first-line treatments for the management of mild-to-moderate acute pain [6,7]. Nonselective NSAIDs inhibit both cyclooxygenase (COX)-1 and COX-2, whereas selective COX-2 NSAIDs have a higher selectivity for COX-2 [8]. The selectivity ratio (COX-1/COX-2 IC50) represents the drug concentration needed for the inhibition of each prostaglandin-synthase isozyme activity by 50% [8]. However, their structure, properties, and ability to inhibit COX-1 and COX-2 isoenzymes determine whether they have a greater antipyretic, anti-inflammatory, or analgesic action [7]. The analgesic effect of NSAIDs is thought to be mediated mainly through the inhibition of COX-2. COX-2 selective NSAIDs inhibit the COX-2 isoenzyme responsible for prostaglandin production, which is involved in the pathway of pain, inflammation, and fever [6,7]. COX-2 selective inhibitors are safe in asthma patients and do not impair platelet function or increase the risk of blood loss in surgeries such as total knee joint replacement, compared to the increased risk of bleeding associated with nonselective NSAIDs [8-10].

Etoricoxib for the management of pain

In 2002, etoricoxib was approved for the acute and chronic treatment of osteoarthritis (OA), rheumatoid arthritis (RA), acute gouty arthritis, ankylosing spondylitis (AS), relief from acute and chronic pain, and primary dysmenorrhea by the European Medicines Agency (EMEA) [8,11]. In 2004, etoricoxib was approved by the Drugs Controller General of India (DCGI) - Central Drugs Standard Control Organisation (CDSCO) in India [9]. Etoricoxib is highly selective for COX-2, with an IC50 ratio of 344 in whole blood and low interaction with COX-1 [8]. Numerous studies have been conducted and have established the use of oral etoricoxib in pain management.

Clarke et al. conducted a study in which nearly 50% pain relief was observed in 66% of the patients administered oral etoricoxib 120mg versus 12% of patients in the placebo group. Further, the use of rescue medication was significantly reduced in patients who received etoricoxib, and the median time to use rescue medication was 20 hours for etoricoxib versus 2 hours for placebo. The safety profile observed with etoricoxib was similar to placebo [12].

Puura et al. conducted a double-blind, randomized, and active placebo-controlled study in patients undergoing elective laparoscopic cholecystectomy. In these patients, oral 120 mg etoricoxib was administered 1.5 hours prior to the surgery, along with 50 mg fentanyl administered through a patient-controlled device for the management of pain. A significantly relevant fentanyl-sparing effect was reported 2-20 hours postoperatively compared to placebo. The study concluded that the need for postoperative opioids was reduced because etoricoxib is a suitable premedication option [13].

Mukhtar et al. conducted a study to evaluate the efficacy and safety of oral etoricoxib 120 mg in reducing postoperative pain associated with minor oral surgeries compared to naproxen 500 mg tablet. The study showed no statistically significant difference in either group after patients were administered drugs 30 minutes before the procedure. The study suggested that etoricoxib can be used as a preemptive medication to reduce pain [14].

Once daily intramuscular (IM) etoricoxib for acute pain in adults: a novel technology

Oral etoricoxib has proven efficacy in delivering analgesia across various settings, including ambulatory care, dental procedures, laparoscopic cholecystectomy, and knee repair [15]. As a safe and effective agent, it demonstrates notable capabilities for managing postoperative pain with minimal adverse effects. It offers advantages over traditional NSAIDs such as naproxen and ibuprofen, including an improved gastrointestinal safety profile and once-daily dosing [8]. However, with limited options available in the toolkit, an alternative like etoricoxib IM is needed to ensure effective and optimum pain relief.

Oral etoricoxib has been extensively studied, but there is a lack of research exploring the use of etoricoxib via alternative routes, specifically IM injection.

IM injection is a well-established and widely used route of drug administration, offering rapid absorption and onset of action [16]. By exploring the effectiveness and safety of etoricoxib administered via IM injection, the treatment options for acute pain management can be potentially expanded. Additionally, IM injection can be valuable for patients who cannot tolerate or absorb oral medications effectively [16].

In 2008, DCGI approved IM etoricoxib injection 90 mg/mL for short-term use in acute painful conditions in adults only. A novel formulation of etoricoxib suitable for administration through the IM route (Nucoxia® injection) has been developed using innovative Ad-Sol technology (advanced solubilization technology), co-solvency, and pH modulation techniques by Zydus Healthcare Ltd. The Ad-Sol technology offers water-insoluble etoricoxib dissolved in water in the presence of citric acid and solubilizers, etoricoxib stability with a blend of excipients, and no irritation at the site of injection because of the hydroalcoholic base.

IM etoricoxib is an addition to the currently available limited parenteral armamentarium. It is also an alternative in cases where oral administration is either not feasible or has been proven to provide inadequate pain relief or trigger tolerability issues with conventional therapies. This study in the real-world context seeks to fill the existing knowledge gap by assessing the practical effectiveness and safety of IM etoricoxib injection in acute pain. To the best of our knowledge, this is the first study in the Indian population that determines the effectiveness and safety of IM etoricoxib 90 mg/mL injection in the management of acute pain in real-time clinical practice.

## Materials and methods

Study design and participants

This was a multicenter, retrospective, observational study to investigate the real-world evidence of IM etoricoxib injection in patients with acute pain in India (outpatient setting). Patients with acute pain in outpatient clinic settings were collected in the Nationwide Etoricoxib Injection Clinical Experience (NICE) registry from November 2022 to April 2023. For this study, medical records from various medical centers that treated patients with etoricoxib injections were selected. Following approval from the site investigator, comprehensive patient-level information encompassing demographic and clinical variables and comorbidities was collected and entered into a case report form. Independent Ethics Committee (IEC) and Institutional Review Board (IRB) approval was sought. The clinical data of 383 patients with acute pain who had received IM etoricoxib injection 90 mg/mL (Nucoxia® injection) as per the treating physician’s discretion were included. In this observational analysis, the data of patients where the visual analogue scale (VAS) at baseline, 30 minutes, and 60 minutes were available were included in the final analysis. The data analysis was carried out after the ethics committee’s approval (Suraksha Ethics Committee dated October 6, 2023).

Efficacy parameters

The primary outcomes of the study include improvement in pain intensity scores at 30 minutes and 60 minutes compared to baseline by the change in the VAS, and the secondary outcomes involving the need for additional analgesia (rescue analgesia) along with an overall assessment of pain relief in IM etoricoxib injection versus currently used NSAIDs.

Safety parameters

Safety was evaluated by recording pain at the injection site after 30 minutes and any allergic reactions or adverse reactions reported post-injection at 30 minutes and 60 minutes.

Statistical analysis

Descriptive statistics were employed to summarize baseline characteristics. Continuous variables were presented as mean and standard deviation, whereas categorical variables were expressed as frequencies and percentages. Appropriate statistical tests were used for data analysis. The data collected were pooled in a Microsoft Excel spreadsheet (Microsoft Corporation, Redmond, Washington) and then transferred for statistical calculations to MedCalc software (v22.013). A p-value of less than 0.05 was considered to be significant for the analysis.

Sample size

This study was considered a post-marketing study because IM etoricoxib injection had already been approved by DCGI in 2008 and is commercially available. A sample size of approximately 400 patients with acute pain was deemed sufficient to collect real-world experience.

## Results

Of the 400 patient forms received, data from 383 patients (males 64.22%, females 35.77%) were included in the final analysis because incomplete forms were excluded from the analysis. The VAS at either of the time points was not available for 17 patients and hence was excluded from the analysis. The mean age of the patients was 48.61 ± 14.20 years, with comorbidities such as diabetes (13.31%), hepatic impairment (12.53%), renal impairment (13.31%), hypertension (21.93%), and cardiovascular diseases (2.34%; Table 1).

**Table 1:** Baseline demographic and clinical characteristics of the study population

Number of patients (N)	Value
Enrolled	400
Dropouts	17
Analyzable	383
Age (years)	
Mean	48.61 ± 14.20
Range	18–89
Gender [no.]	
Male	246
Female	137
Comorbid conditions (no.)	
Renal impairment	51
Hepatic impairment	48
Diabetes	73
Hypertension	84
Cardiovascular diseases	9

Indications for which etoricoxib injection was used

**Figure 1:**
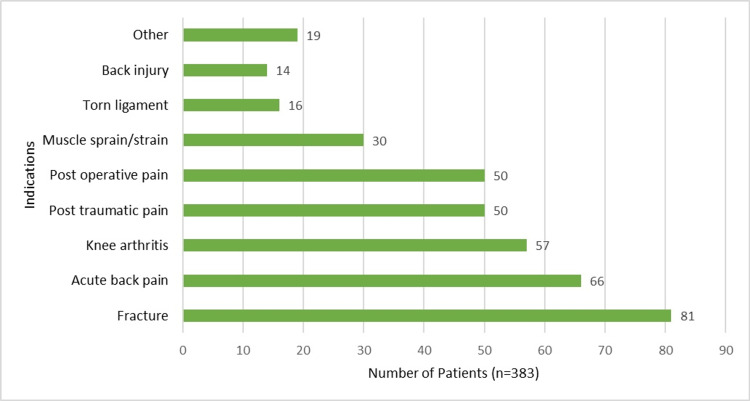
IM etoricoxib for management of acute painful conditions

IM etoricoxib in 383 patients was used for the management of acute pain associated with arthritis, fracture-related pain, post-traumatic pain, back pain and injury, muscle strain or sprain, torn ligament, and postoperative pain. A few patients suffered from other ailments such as knee synovitis, calcaneal spurs, and tibial abscess pain (Figure 1).

Effectiveness of etoricoxib at 30 and 60 minutes

**Figure 2:**
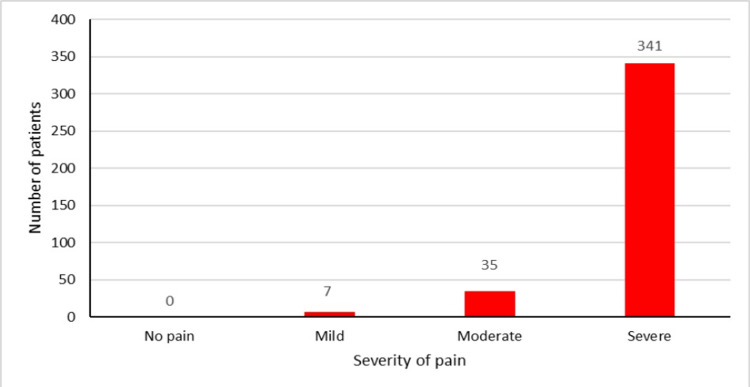
VAS score of patients at 0 minutes VAS: visual analogue scale

The VAS was classified as no pain (0), mild pain (1-3), moderate pain (4-6), and severe pain (7-10). At baseline (0 minutes), 98.17 % (n = 376) of patients experienced moderate-to-severe pain (Figure 2).

**Figure 3:**
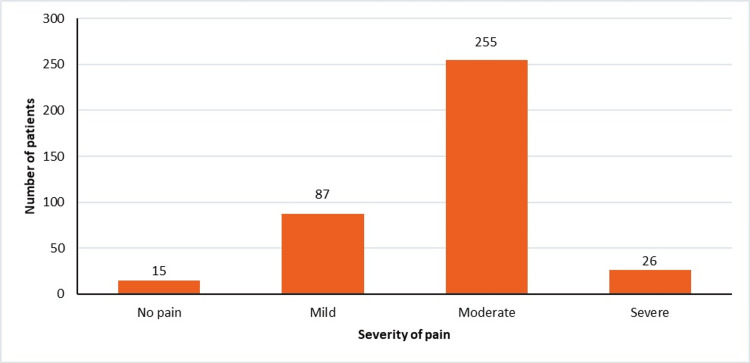
VAS score of patients at 30 minutes VAS: visual analogue scale

At 30 minutes, only 6.78% (n = 26) of patients complained of severe pain, and 3.91% (n = 15) of the patients became pain-free, whereas 22.71% (n = 87) of patients had mild pain only (Figure 3).

**Figure 4:**
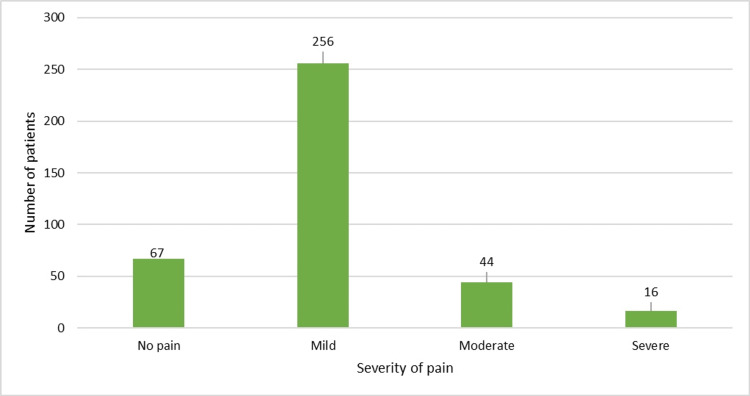
VAS score of patients at 60 minutes

Lastly, at 60 minutes, 17.49% (n = 67) of the patients had become pain-free, 66.84% (n = 256) of the patients had only mild pain post-IM etoricoxib injection, and 15.66% (n = 60) of the patients were left with moderate-to-severe pain (Figure 4).

The mean VAS at baseline was 7.89 ± 1.44. Mean VASs at 30 minutes and 60 minutes post-IM etoricoxib injection were 4.23 ± 1.80 and 2.14 ± 1.78, respectively (Table 2). After the administration of IM etoricoxib injection, at the end of 30 minutes, the mean VAS decreased significantly by 46.38% (mean ± SD, p < 0.0001). At the end of one hour, the mean VAS significantly decreased to 72.8% (p < 0.0001). The improvement in the VAS was statistically significant from 30 to 60 minutes, with the mean VAS decreasing by 49.4% (p < 0.0001).

**Table 2:** VASs at different time points during the study period *p < 0.0001 as compared to baseline and at 30 minutes compared to 60 minutes VAS: visual analogue scale

Observational points	Mean VAS (mean ± SD)
Baseline (0 minutes)	7.89 ± 1.44
30 minutes	4.23 ± 1.80*
60 minutes	2.14 ± 1.78*

Most responders, 70.23% (n = 269), opined that the etoricoxib injection was better than their currently used NSAID injections for pain relief. Approximately 27.67% (n = 106) opined that etoricoxib injection was equal to currently used NSAID injections for pain relief, whereas only 2.09% (n = 8) reported that the etoricoxib injection was worse than their currently used NSAID injections.

A minority of patients, 12.79% (n = 49), required rescue analgesias such as oral paracetamol, oral NSAIDs, or injection diclofenac post-IM etoricoxib owing to either severe pain or pain not adequately relieved by the IM injection.

Safety of etoricoxib

**Figure 5:**
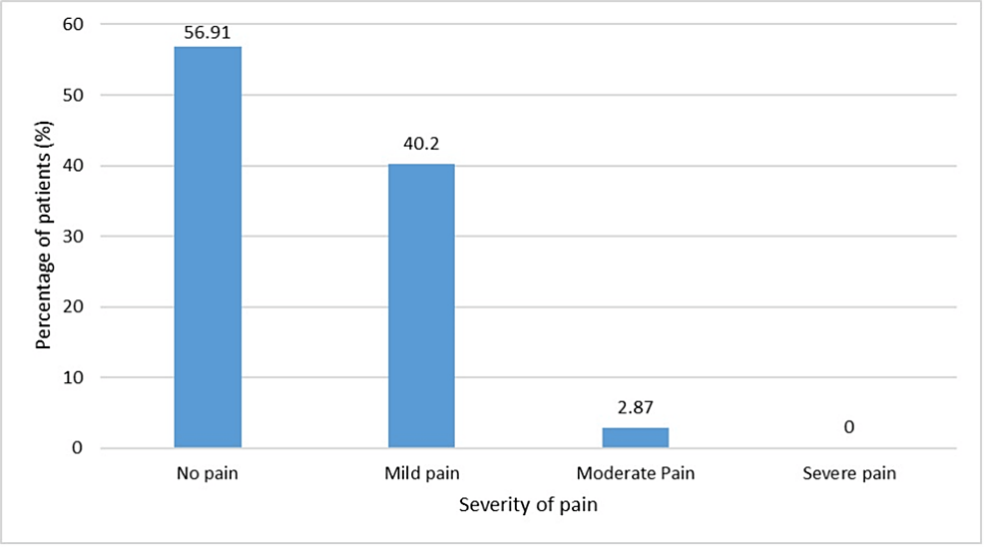
Pain at the site of injection after IM etoricoxib injection IM: intramuscular

More than half of the patients, 56.91% (n = 218), had no pain at the site of the injection. None of the patients had severe pain post-injection (Figure 5). Allergic reactions post-injection were reported by 4.44% (17/383) of patients. IM etoricoxib injection was very well tolerated, given that 98.69% of the patients did not experience or report any adverse events. Only 1.3% of patients had adverse events like burning and tingling at the site of injection. None of the patients had any serious adverse events post-etoricoxib injection.

## Discussion

Recent guidelines advocate for multimodal analgesia that combines diverse medications and techniques with non-pharmacological interventions to treat acute pain. Benefits include accelerated recovery activities and reduced morbidity, mortality, and costs. The goal of combining non-opioid analgesics with opioids is to enhance pain relief and minimize adverse events, but guidelines emphasize routine inclusion of non-opioid options [17].

NSAIDs have proven efficacy in the management of acute and chronic pain. The efficacy of selective COX-2 inhibitors is similar to that of nonselective NSAIDs but with better GI tolerability. Etoricoxib, a highly selective COX-2 inhibitor, has demonstrated anti-inflammatory, analgesic, and antipyretic activities in acute and chronic pain. Etoricoxib is used to treat inflammatory diseases such as OA, RA, ankylosing spondylitis, acute gout, and acute episodes of chronic inflammatory conditions [8].

Etoricoxib, employed in pain therapy, exhibits distinctive features. It exhibits a chondroprotective effect by reducing joint inflammation and proinflammatory cytokine levels, which is particularly beneficial for osteoarthritis. Etoricoxib’s compatibility in combination therapy highlights its additive and synergistic effects, ensuring stability in both analgesic and anti-inflammatory outcomes. Overall, its pharmacokinetic profile not only enhances analgesic potency but also sustains its anti-sensitizing properties, offering a comprehensive approach to pain management. The multifaceted impact of etoricoxib is discernible through its numerous effects. Table 3 highlights the most significant among them [11].

**Table 3:** Comprehensive Impact of etoricoxib in inflammatory pain

Features of etoricoxib	Clinical significance
Mechanism of action	In addition to selective COX-2 activity, it has additional non-cyclooxygenase activities such as antisensitizing effects, which reduce the risk of chronic pain, anti-inflammatory effects reducing the concentration of pro-inflammatory cytokines which in turn reduces the destruction of articular cartilage
PK/PD of etoricoxib	The mean peak plasma concentration (C_max_) of etoricoxib was 95–2186 ng/mL over the dose range of 5–120mg. The time to reach C_max_ (T_max_) was comparable over the same dose range at approximately 1 hour. A large volume of distribution, around 120 L, affects the peripheral effect of the action and is particularly important in patients with comorbid obesity
Effect on cytochrome P450 isoenzymes	Reduced pharmacokinetic interactions as etoricoxib is metabolized by CYP3A4 but has no inhibitory effect on cytochrome P450 isoenzymes therefore limited risk of drug–drug interactions
Stability of the analgesic effect in inflammatory pain	Stable analgesic and anti-inflammatory effect due to long half-life of up to 24 hours reducing the risk of pain fluctuation
Side-effect profile	COX-2 inhibitors are known to have a safer gastrointestinal safety profile than traditional non-selective NSAIDs. Etoricoxib reduces the risk of damage to the upper gastrointestinal tract due to higher COX-2 selectivity, limiting the need to use drugs from the group of proton pump inhibitors

Multiple clinical studies have documented the safety and efficacy of oral etoricoxib in managing pain. A systematic review of 10 clinical studies showed that oral etoricoxib did not increase cardiovascular events compared to placebo or NSAIDs during a 12-week period, documenting etoricoxib as an alternative to short-term treatment options [18]. Oral etoricoxib 120 mg, administered one hour before surgery, also significantly reduced the postoperative pain score, improved sleep, and patient satisfaction, and reduced postoperative opioid requirements [15].

The current study evaluated patients receiving IM etoricoxib injection (Nucoxia®) and highlighted the effectiveness and safety of etoricoxib in managing acute pain. There was a significant improvement in the VAS after IM etoricoxib injection. Of the 383 patients, 98.17% had moderate-to-severe pain at baseline. The percentage of patients with severe pain reduced to only 6.78% and 4.17% at 30 minutes and 60 minutes, respectively. In this real-world evidence, IM etoricoxib injection was well tolerated, as most patients reported no pain or mild pain at the injection site (97.1%). Most participating clinicians (97.91%) opined that IM etoricoxib injection was better or comparable to currently used NSAID injections based on their overall assessment.

The findings suggest that IM etoricoxib injection 90 mg/mL is effective, well-tolerated, and devoid of any significant untoward side effects. These favorable outcomes further support the use of IM etoricoxib injection in managing acute pain.

This retrospective study's strengths lie in its thorough examination of subject demographics and baseline clinical characteristics. Conducted in real-world settings, the study offers valuable insights into the practical effectiveness of a novel IM etoricoxib injection. However, the study also has several limitations that must be acknowledged, such as the absence of randomization, unlike controlled prospective studies, and the absence of monitoring for 24 hours in an outpatient setting. Future studies could focus on observing the improvement in the VAS over 24 hours and the quality of life of the patients post-IM etoricoxib injection use.

## Conclusions

In conclusion, this real-world evidence, comprising 383 patients at 42 centers across India, showcased that the innovative and novel formulation of IM etoricoxib 90 mg/mL injection (Nucoxia®) appears to be effective and well-tolerated in the management of acute pain. IM etoricoxib is found to be efficacious and safe in the management of acute pain across multiple clinical conditions. Most participating clinicians found IM etoricoxib to be better or comparable to their currently used NSAID injections. Overall, this study provides valuable insights into the real-world effectiveness and safety of IM etoricoxib injections, suggesting it is a promising therapy for the management of acute pain for optimal patient benefit.
